# Comparison of Clinical, Metabolic and Hormonal Effects of Metformin Versus Combined Therapy of Metformin With Myoinositol Plus D-Chiro-Inositol in Women With Polycystic Ovary Syndrome (PCOS): A Randomized Controlled Trial

**DOI:** 10.7759/cureus.15510

**Published:** 2021-06-07

**Authors:** Anupama Bahadur, Hitanshi Arora, Anoosha K Ravi, Manisha Naithani, Yogesh Bahurupi, Jaya Chaturvedi, Megha Ajmani, Rajlaxmi Mundhra

**Affiliations:** 1 Obstetrics and Gynecology, All India Institute of Medical Sciences, Rishikesh, Rishikesh, IND; 2 Biochemistry, All India Institute of Medical Sciences, Rishikesh, Rishikesh, IND; 3 Community & Family Medicine, All India Institute of Medical Sciences, Rishikesh, Rishikesh, IND

**Keywords:** polycystic ovary syndrome (pcos), insulin resistance, inositol, myoinositol, d-chiro-inositol, acne, menstrual cycle irregularity

## Abstract

Objective

To compare the effects of metformin alone versus combined therapy of metformin with myoinositol (MI) plus D-chiro-inositol (DCI) in women with polycystic ovary syndrome (PCOS).

Materials and methods

This is a prospective, non-blinded randomized controlled trial conducted in newly diagnosed PCOS women aged 18 to 45 years. Group I received metformin 500 mg twice a day orally for 6 months while group II received metformin 500 mg twice a day orally along with MI 550 mg plus DCI 150 mg twice daily orally for six months. The primary outcome was a change in clinical, metabolic and hormonal parameters of the two groups from baseline to the end of six months of treatment.

Results

A total of 72 patients were randomized into two groups of 36 patients each. Statistically, a significant difference was seen in terms of mean global acne score (p=0.004) and cycle regularity (p=0.034) after six months of treatment in group II. A significant difference in values of luteinizing hormone (LH) (p=0.002), luteinizing hormone/follicle-stimulating hormone (LH/FSH) ratio (p=0.007), mean cholesterol (p=0.040), mean high-density lipoprotein (HDL) (p=0.049), mean low-density lipoprotein (LDL) (p=0.0001) and postprandial insulin (p=0.005) was also seen in group II at the end of treatment duration. No significant difference was seen between the two groups in terms of mean FSH, mean testosterone, mean dehydroepiandrosterone sulfate (DHEAS), mean triglyceride, mean fasting and postprandial blood sugar, fasting insulin and homeostatic model assessment of insulin resistance (HOMA-IR) index.

Conclusion

Combined therapy with metformin and MI plus DCI in women with PCOS and insulin resistance seems promising with the need for further studies with a greater sample size to evaluate the efficacy of this treatment.

## Introduction

Polycystic ovary syndrome (PCOS) is one of the most common endocrine disorders affecting about 4% to 8% of women of reproductive age [1]. Diagnosis is primarily clinical with Rotterdam criteria being the most commonly followed criteria, which require the presence of two out of the three criteria including oligo and/or anovulation, clinical and/or biochemical signs of hyperandrogenism and polycystic ovaries on ultrasound [2].

Hyperandrogenism and insulin resistance are the key features of PCOS. Insulin resistance results from post binding abnormality in insulin receptor-mediated signal transduction [3]. It leads to impaired glucose tolerance, hypertension and dyslipidemia and thereby risks of coronary artery disease. Hence women with PCOS should be screened for metabolic syndrome and glucose intolerance.

To prevent the long-term health consequences of PCOS, besides lifestyle modifications [4], the use of insulin-sensitizers has been proposed, of which metformin is commonly being used [5]. As per Cochrane review, it was found that metformin significantly decreases fasting glucose and insulin levels as well as insulin levels after oral glucose administration as compared to placebo [6]. Androgen levels have also shown to be reduced with metformin but there is no evidence that it reduces hirsutism [7].

Recently promising results have been seen following the use of inositol stereoisomers such as Myo-inositol (MI) and D-chiro-inositol (DCI) acting like insulin mediators. They are hexahydroxycyclohexane members of the family of nine stereoisomeric inositols commonly found in fruits and beans [8]. MI part of the B-complex family is a natural insulin sensitizer. It is a component of membrane phospholipids and produces inositol phosphate as a second messenger [9]. DCI is the main inositol found in fat, muscle and liver, and is responsible for glycogen synthesis. At the ovarian level, MI acts as an important constituent of the follicular microenvironment thereby playing a key role in oocyte development. DCI has an indirect effect on the ovary by reducing hyperinsulinemia and increasing glycogen synthesis. It is also responsible for insulin-mediated testosterone synthesis. MI improves ovarian function, supports follicle-stimulating hormone (FSH) signaling and thus decreases luteinising hormone/follicle-stimulating hormone (LH/FSH) ratio, increases sex hormone-binding globulin (SHBG) and reduces serum total and free testosterone [8].

The available literature has shown that inositol has a beneficial effect on PCOS due to its insulin sensitising activity [10-12]. Based on this finding, we aimed to investigate the effects of the most commonly used therapy, i.e., metformin, used alone versus combined therapy of metformin with MI plus DCI in women with PCOS.

## Materials and methods

This was a prospective, non-blinded randomized controlled trial, conducted in Obstetrics and Gynaecology Department of a single institution over a period of 18 months from December 2016 to May 2018. Women in the reproductive age group 18 to 45 years with newly diagnosed PCOS according to Rotterdam criteria and those willing to participate in the study and follow up were included after written informed consent. Patient already on other drugs for the treatment of PCOS like oral contraceptive pills, having deranged kidney or liver function tests, uncontrolled thyroid disorders, hyperprolactinemia, known hypersensitivity to metformin or MI plus DCI and those with any other endocrinological disorder like congenital adrenal hyperplasia, Cushing’s syndrome or androgen-secreting tumors were excluded from the study.

A detailed clinical history including menstrual history, obstetric history and family history of diabetes mellitus was recorded. Hirsutism (0-36) by modified Ferriman Galway (mFG) score, acne score and blood pressure were noted for all patients. Anthropometric measurements including height (in cm), weight (in kg), hip circumference (in cm) and waist circumference (in cm) were measured and body mass index (BMI in kg/m^2^) was calculated. Overweight was defined using the Asian BMI range of 23.0-26.9 kg/m^2^, while ≥27 kg/m^2^ were considered obese. Detailed gynaecological examination including bimanual examination except in adolescents and unmarried women was done to rule out any local pelvic pathology. All study subjects underwent biochemical and hormonal assessments during their respective follicular phases (2-5 days of the menstrual cycle). Fasting, 0 and 2-hour blood samples for 75 gm oral glucose tolerance test (OGTT), fasting and 2-hour postprandial samples for serum insulin and samples for lipid profile, FSH, LH, testosterone and dehydroepiandrosterone sulfate (DHEAS) were collected on day 2/3 of the menstrual cycle.

Patients were randomized into two groups based on a computer-generated randomization table. Group I received metformin 500 mg twice a day orally for six months while group II received metformin 500 mg twice a day orally along with MI 550 mg plus DCI 150 mg twice a day orally for six months. All the measurements were repeated at the end of six months and a note of clinical assessment was made.

The primary outcome of the study was to analyze the change in clinical, metabolic and hormonal parameters of the two groups from baseline to the end of six months of treatment. Improvement in clinical parameters was noted in terms of menstrual cycle irregularity, changes in mFG score, global acne score, waist circumference, hip circumference, waist:hip ratio and BMI. Improvement in metabolic and hormonal parameters included changes in lipid profile, fasting and postprandial blood sugar, fasting and postprandial insulin, LH/FSH ratio, serum testosterone, DHEAS and homeostatic model assessment of insulin resistance (HOMA-IR) index.

Statistical analysis

The data were represented as mean±SD. The continuous data were compared by applying the Student t-test. The categorical data were compared by applying Pearson’s chi-square test or Fisher’s exact test wherever necessary. P < 0.05 was considered significant. All statistical analysis was carried out using Stata 12.0 (Stata Corp LP, College Station, TX, USA).

Ethical clearance was obtained from the institutional ethics committee AIIMS/IEC/16/138. This trial was registered with the Clinical Trial Registry of India CTRI/2018/05/013977. There was no funding required.

## Results

**Figure 1:**
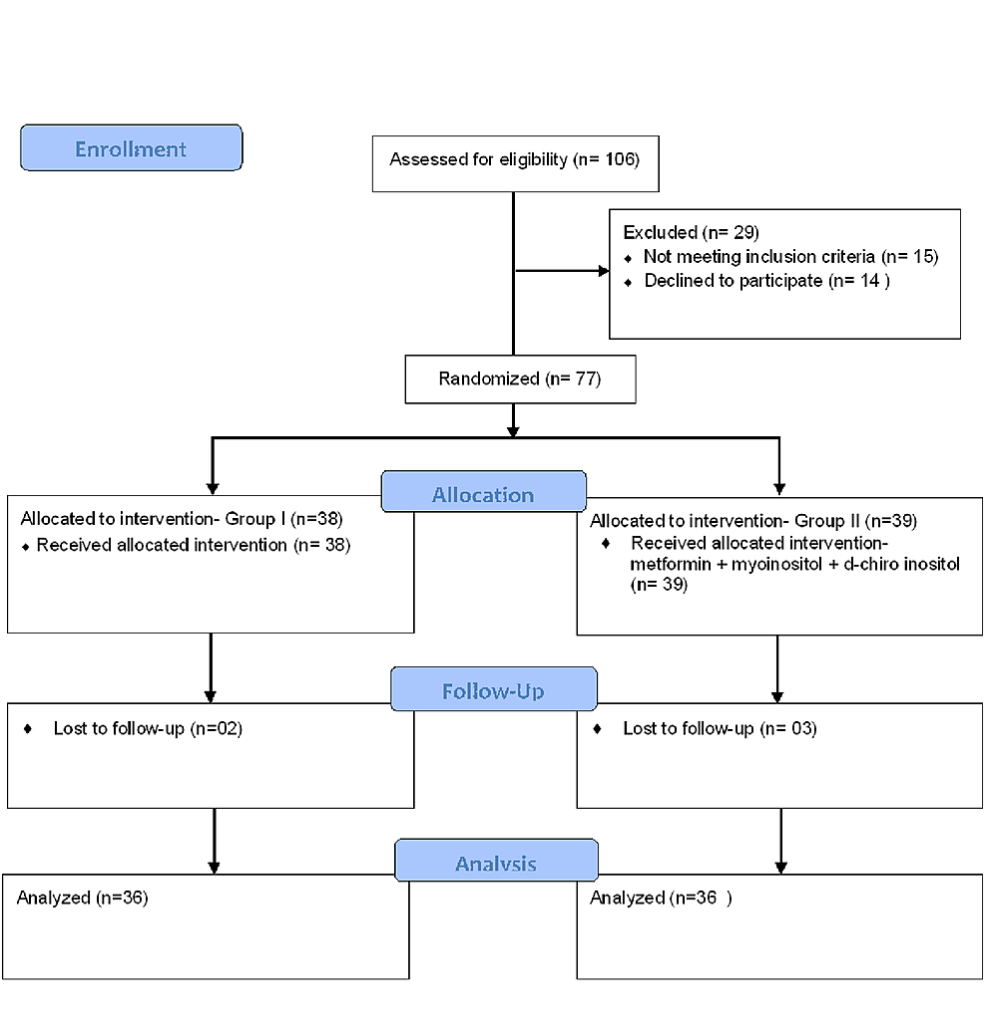
Study population flow chart

A total of 106 patients were assessed for eligibility out of which 29 patients were excluded (Figure 1). The remaining 77 patients were selected and randomized into two groups. Two patients in group I and three patients in group II were lost to follow up. Thus, 36 patients in each group were analysed based on per protocol analysis.

Both the groups were comparable in baseline clinical characteristics as shown in Table 1. Table 2 shows the comparison of clinical parameters including anthropometric measurements between the two groups at baseline and six months. The difference between the two groups in terms of mean global acne score after six months of treatment was statistically significant (p=0.004). At baseline, cycle irregularity was seen in all the study subjects of both groups. At six months follow up, 61.1% of patients in group II had attained normal cycles whereas only 36.1% of group I had attained regular cycles (p=0.034).

**Table 1:** Comparison of baseline clinical characteristics of two groups Data presented as mean±SD and n (%) ^#^by applying Fisher's Exact Test ^##^by applying Pearson’s chi-square test BMI: body mass index

Sl. no.	Baseline clinical characteristic	Group I	Group II	P value
1	Age, years	21.89±4.23	23.78±4.46	0.06
2	BMI, kg/m^2^	23.43±4.75	25.29±4.13	0.080
3	Family history of diabetes	3 (8.3)	4 (11.1)	1.000^#^
4	Oligomenorrhea	36 (100)	33 (91.7)	0.239^#^
5	Acne	9 (25)	7 (19.4)	0.571 ^##^
6	Hirsutism	11 (30.6)	6 (16.7)	0.165 ^##^

**Table 2:** Comparison of clinical parameters of two groups at baseline and at six months Data presented as mean±SD ^#^Date presented as n% *Mann-Whitney test applied **Pearson’s chi-square test applied mFG: modified Ferriman Galway score, BMI: body mass index

Clinical characteristic	Group I baseline	Group II baseline	P-value	Group I at six months	Group II at six months	P-value
mFG score	7.14±4.75	8.67±4.60	0.170	5.47±3.22	4.86±2.70	0.174*
Global acne score	5.14±3.63	5.11±3.51	0.816	4.41±2.59	2.80±1.39	0.004*
BMI, kg/m^2^	23.43±4.76	25.29±4.13	0.076	23.36±4.08	23.34±3.14	0.993*
Waist circumference, cm	82.07±14.02	87.44±9.40	0.060	81.11±12.68	82.81±8.20	0.503^*^
Hip circumference, cm	97.44±10.03	102.49±7.49	0.018	95.17±8.99	97.33±7.55	0.272^*^
Waist:hip ratio	0.84±0.08	0.85±0.06	0.446	0.85±0.08	0.85±0.05	0.948^*^
Cycle irregularity^#^	100.0%	100.0%	-	63.9%	38.9%	0.034^**^

At the end of six months of treatment duration, there was a significant decrease in LH and LH:FSH ratio in group II compared to group I (Table 3). Also, there was a significant improvement in cholesterol, HDL, LDH and postprandial insulin values with six months of treatment with combined therapy compared to metformin alone (Table 4).

**Table 3:** Comparison of hormonal parameters of two groups at baseline and at six months Data presented as mean±SD LH: luteinizing hormone, FSH: follicle-stimulating hormone, DHEAS: dehydroepiandrosterone sulfate

Parameter	Group I baseline	Group II baseline	P-value	Group I at six months	Group II at six months	P-value
LH, IU/L	9.19±5.77	8.52±4.99	0.597	9.09±4.53	6.06±2.24	0.002*
FSH, IU/L	6.49±2.31	5.78±1.73	0.389	6.43±2.07	5.64±1.23	0.054
LH:FSH	1.47±0.94	1.51±0.97	0.888	1.39±0.43	1.11±0.44	0.007*
Total testosterone, ng/dL	49.26±20.80	49.54±20.20	0.955	54.56±18.79	47.55±17.49	0.106
DHEAS, µg/dL	192.88±98.59	208.44±98.82	0.260	191.37±88.96	188.62±97.81	0.770

**Table 4:** Comparison of metabolic parameters of two groups at baseline and at six months Data presented as mean±SD HDL: high-density lipoprotein, LDL: low-density lipoprotein, FBS: fasting blood sugar, PPBS: postprandrial blood sugar. HOMA-IR: homeostatic model assessment of insulin resistance

Parameter	Group I baseline	Group II baseline	P-value	Group I at six months	Group II at six months	P-value
Cholesterol, mg/dL	149.14±36.94	159.25±38.79	0.261	146.75±36.37	131.58±23.99	0.040*
Triglyceride, mg/dL	91.78±22.76	114.64±70.81	0.176	96.94±26.90	95.61±38.46	0.389
HDL, mg/dL	39.75±8.52	42.14±7.72	0.217	41.53±6.38	47.25±15.92	0.049
LDL, mg/dL	89.68±35.12	98.73±30.67	0.313	106.16±22.78	85.89±19.84	0.0001*
FBS, mg/dL	88.28±9.97	87.42±10.74	0.725	87.50±8.25	84.58±5.63	0.084
PPBS, mg/dL	104.83±19.65	109±17.59	0.346	103.89±15.76	102.03±17.22	0.634
Fasting insulin, mIU/L	15.84±10.02	20.33±12.05	0.166	15.04±8.19	14.68±9.16	0.858
Postprandial insulin, mIU/L	92.66±94.99	81.63±70.16	0.978	57.82±37.52	35.30±18.94	0.005*
HOMA-IR	3.45±2.20	4.44±2.87	0.189	3.23±1.73	3.08±2.05	0.746

## Discussion

Insulin resistance plays a key role in the pathogenesis of PCOS and hence decreasing circulating insulin levels serves as a therapeutic target in them [13]. While Metformin is the classical and most frequently used drug for the treatment of PCOS, recently there is a focus on MI [14,15]. Current evidence suggests that MI could improve metabolic profile in PCOS by acting as an insulin sensitising agent [16,17]. Different studies have used 1-4 g per day dose of MI in different research settings and the ideal dose is yet to be defined [18-20]. To the best of our knowledge, no study has been done to study the effect of combined metformin and MI plus DCI to evaluate changes in metabolic and hormonal parameters in PCOS in comparison to metformin alone.

Angik et al. [10], in their study on 100 PCOS women, compared metabolic and hormonal effects of MI versus metformin. They observed that there was a statistically significant reduction in the mean mFG score of hirsutism after six months in both the groups but on comparing the two groups, the reduction was not significant (p=0.813) similar to what was observed in our study (p=0.174). The difference between the two groups in terms of cycle regularity was statistically significant (p=0.002). Cycle regularity was also noted in our study (p=0.034).

In another study by Fruzzetti et al. [11], the authors compared the effects of metformin and MI on the clinical and metabolic features of 50 women with PCOS. A slight improvement of hirsutism was reported in 12% and 20% of patients in the metformin and MI groups, respectively. No statistically significant difference was seen in acne score between the two groups at the end of six months. In contrast, our study showed a reduction in mean acne score from baseline to six months (p=0.004).

Benelli et al. [12] conducted a study on 46 patients affected by PCOS. They randomly, assigned the sample in two groups, A and B, treated, respectively, with MI plus DCI in a ratio of 40:1 and with folic acid (placebo) for six months. A statistically significant reduction of LH, free testosterone, fasting insulin and HOMA-IR index was seen in the group treated with the combined therapy of MI plus DCI as compared to placebo. In the present study, combined therapy resulted in a significant difference in values of LH and postprandial insulin levels but not in HOMA-IR index.

The strength of the present study lies in the well-designed study model to analyze the benefits of the proposed combined therapy in patients with PCOS. The limitation of the study is that it has a small sample size. Therefore more studies with a greater sample size are required to further evaluate the efficacy and safety of inositols with or without metformin.

## Conclusions

MI and DCI are relatively newer agents in the treatment of PCOS. This study was conducted to evaluate the clinical, metabolic and hormonal effects of combined therapy of metformin with MI plus DCI versus more traditionally used treatment with metformin. Our study has shown a synergistic effect of metformin in combination with MI plus DCI in women with PCOS and insulin resistance in terms of improvement in cycle irregularity, global acne score, LH levels, LH:FSH ratio, lipid profile including cholesterol, HDL and LDL levels and postprandial insulin. Thus, combined therapy may have a therapeutic and promising role in women with PCOS. However, more studies with a greater sample size are required to further evaluate the efficacy of this treatment.
